# Efficacy and Safety of Vonoprazan-Based versus Proton Pump Inhibitor-Based Triple Therapy for* Helicobacter pylori* Eradication: A Meta-Analysis of Randomized Clinical Trials

**DOI:** 10.1155/2019/9781212

**Published:** 2019-05-09

**Authors:** Qiu-Ju Lyu, Qiang-Hong Pu, Xian-Fei Zhong, Jin Zhang

**Affiliations:** ^1^Department of Endocrinology, People's Hospital of Leshan, Sichuan 614000, China; ^2^Department of Pharmacy, People's Hospital of Leshan, Sichuan 614000, China; ^3^Department of Gastroenterology, People's Hospital of Leshan, Sichuan 614000, China

## Abstract

**Aims:**

To compare the efficacy and safety of vonoprazan-based versus proton pump inhibitor (PPI)-based triple therapy in the eradication of* Helicobacter pylori*.

**Methods:**

We performed a systematic search in PubMed, Embase, and the Cochrane Library databases for relevant randomized controlled trials up to March 2019. Studies were included if they compared the efficacy and safety of* H. pylori* eradication of vonoprazan-based and PPI-based triple therapy.

**Results:**

Three studies with 897 patients were evaluated in this meta-analysis. The* H. pylori *eradication rate of vonoprazan-based triple therapy was higher than that of PPI-based triple therapy as first-line regimens (intention-to-treat analysis: pooled eradication rates, 91.4% vs 74.8%; odds ratio [OR], 3.68; 95% confidence interval (CI): [1.87–7.26];* P*<0.05). The incidence of adverse events in vonoprazan-based triple therapy was lower than that in PPI-based triple therapy (pooled incidence, 32.7% vs 40.5%; OR, 0.71; 95%CI: [0.53–0.95];* P*<0.05).

**Conclusions:**

Efficacy of vonoprazan-based triple therapy is superior to that of PPI-based triple therapy for first-line* H. pylori *eradication. Additionally, vonoprazan-based triple therapy is better tolerated than PPI-based triple therapy.

## 1. Introduction

In 2015, over four billion people were estimated to be infected with* Helicobacter pylori *(*H. pylori*) worldwide [1].* H. pylori* infection causes many gastrointestinal diseases, such as peptic ulcer, chronic gastritis, gastric cancer, and gastric mucosa-associated lymphoid tissue (MALT) lymphoma [2–4].

For* H. pylori *infection, the recommended first-line eradication regimen was proton pump inhibitor (PPI)-based triple therapy, which consisted of a PPI plus amoxicillin and clarithromycin or metronidazole [5, 6]. However, the eradication rate of PPI-based triple therapy has been declining in recent years, owing to increased* H. pylori *resistance to clarithromycin and metronidazole [7, 8]. In China,* H. pylori *resistance rates for clarithromycin and metronidazole were 63.4% and 52.6%, respectively [9]. Currently, bismuth-containing quadruple therapy and concomitant therapy (PPI and three antibiotics) are recommended as first-line options in China and Taiwan, where the prevalence of primary clarithromycin resistance is >15% [10–12].

Vonoprazan is a new oral acid suppressant, which, like PPIs, belongs to a group of H^+^-K^+^ ATPase inhibitors. However, unlike PPIs, vonoprazan is a reversible H^+^-K^+^ ATPase inhibitor [13]. Vonoprazan has been approved to treat* H. pylori* infection, gastroduodenal ulcer, and reflux esophagitis in Japan since February 2015, but it has still not been approved by Chinese, American, and European agencies. Vonoprazan has a potency of H^+^-K^+^ ATPase inhibition approximately 350 times higher than that of PPIs and has a faster, stronger, and longer-lasting acid-inhibitory effect than PPIs have in clinical trials [14–16]. Therefore, vonoprazan was expected to improve the* H. pylori *eradication rate compared with PPIs. Recently, several meta-analyses showed that vonoprazan-containing triple therapy was superior to PPI-containing triple therapy [17–19]. However, these meta-analyses included mostly nonrandomized controlled trials (NRCTs) and were likely to have reported less accurate or robust results when compared to analyses that included only RCTs. Therefore, we performed a meta-analysis including only RCTs to assess the efficacy and safety of vonoprazan-based and PPI-based triple therapy for* H. pylori *eradication.

## 2. Methods

### 2.1. Criteria for Considering Studies for This Meta-Analysis

#### 2.1.1. Types of Studies

Only RCTs that compared vonoprazan-based versus PPI-based triple therapy as first-line regimens for* H. pylori* eradication were included. The language of the studies was restricted to English. The following were excluded: (1) animal studies; (2) other study designs (letters, case reports, editorials, commentaries and reviews, etc.); (3) studies with incomplete data such as abstract-only publications; and (4) studies with duplicate data.

### 2.2. Types of Participants

#### 2.2.1. Inclusion Criteria

RCTs were eligible for inclusion if enrolled participants were diagnosed as positive for* H. pylori* (with one or more confirmatory tests) on the basis of the urea breath test (UBT), rapid urease test, culture, and stool* H. pylori *antigen [20]. Participants had to be naïve to* H. pylori* eradication treatment.

#### 2.2.2. Exclusion Criteria

RCTs were excluded if enrolled participants were diagnosed as* H. pylori*-positive solely on the basis of serology or polymerase chain reaction (PCR) or if the participants had previously been treated with any eradication therapy[20].

### 2.3. Types of Interventions

Vonoprazan-based triple therapy consisted of vonoprazan and two antibiotics, and PPI-based triple therapy consisted of a PPI and two antibiotics. Antibiotic types and doses and duration of treatment were similar between the vonoprazan-based and PPI-based regimens.

### 2.4. Types of Outcome Measures

Relevant trials were included that assessed the following outcomes: (1) Eradication rate: intention-to-treat (ITT) and per-protocol (PP) analyses. Trials were eligible if* H. pylori *eradication was confirmed by UBT or stool* H. pylori *antigen, at least 4 weeks after completion of treatment. (2) Incidence of adverse events (ITT analysis). Adverse events included diarrhea, dysgeusia, and any type of adverse events.

### 2.5. Search Strategy

#### 2.5.1. Electronic Searches

We performed a systematic search of PubMed, Embase, and the Cochrane Library databases for relevant RCTs up to March 18, 2019. The following terms were used: (“vonoprazan” or “takecab” or “TAK438” or “TAK-438” or “potassium-competitive acid blocker”) and (“proton pump inhibitors” or “omeprazole” or “lansoprazole” or “pantoprazole” or “rabeprazole” or “esomeprazole” or “ilaprazole” or “dexlansoprazole” or “dexrabeprazole” or “tenatoprazole”) and (“*Helicobacter pylori*” or “*Campylobacter pylori*”) and (“randomized controlled trial”). The detailed search strategies are shown in Appendix S1. The language of the studies was restricted to English.

#### 2.5.2. Searching Other Resources

Two investigators (Qiang-Hong Pu and Qiu-Ju Lyu) carefully screened the reference lists of the retrieved articles to identify additional studies.

### 2.6. Data Collection and Analysis

#### 2.6.1. Selection of Studies

First, we excluded the duplicate studies using Endnote software Version X8 and manual screening (author, title, journal, publication year, journal volume, and issue). Second, we excluded the irrelevant studies through examining the title and abstract of articles. Lastly, we examined the full text of the remaining studies according to the inclusion and exclusion criteria. Two investigators (Qiang-Hong Pu and Qiu-Ju Lyu) independently assessed the studies identified by the literature search. Any disagreement was resolved in a consensus meeting with all the authors.

#### 2.6.2. Data Extraction

Two investigators (Qiang-Hong Pu and Qiu-Ju Lyu) independently extracted data using a predesigned data extraction form, according to the method developed by Li and Jung [18, 19]: first author, publication year, country, eradication regimens (dosage and frequency of vonoprazan, PPIs and antibiotics), duration of treatment, confirmative test for eradication, eradication rate (ITT and PP analyses), and adverse events.

### 2.7. Assessment of Risk of Bias in Included Studies

Two investigators (Qiang-Hong Pu and Qiu-Ju Lyu) independently assessed the risk of bias of included RCTs using the Cochrane Risk of Bias assessment tool [21]: (1) how the random sequence was generated; (2) how patient allocation was concealed; (3) blinding of the patients and researchers; (4) blinding of outcome assessment; (5) whether there were incomplete outcome data; (6) whether there was selective outcome reporting; and (7) other potential biases.

### 2.8. Assessment of Heterogeneity

Heterogeneity was evaluated by Cochrane's Q test, which was considered statistically significant for heterogeneity if* P* was <0.1, and I^2^ statistics, for which 30%–60% and 60%–90% suggested moderate and substantial heterogeneity, respectively.

### 2.9. Assessment of Reporting Biases

Since there were <10 included studies, the publication bias (test for funnel plot asymmetry) was not evaluated.

### 2.10. Data Synthesis and Statistical Analysis

Meta-analyses were conducted using RevMan version 5.3 (Cochrane Collaboration, Copenhagen, Denmark) with random-effect model by default. All statistical tests were two-tailed;* P*<0.05 was considered statistically significant in all tests (except for the heterogeneity test), and pooled odds ratios (ORs) with 95% confidence interval (CI) were calculated.

## 3. Results

### 3.1. Studies Selection and Characteristics of Included Studies

**Figure 1:**
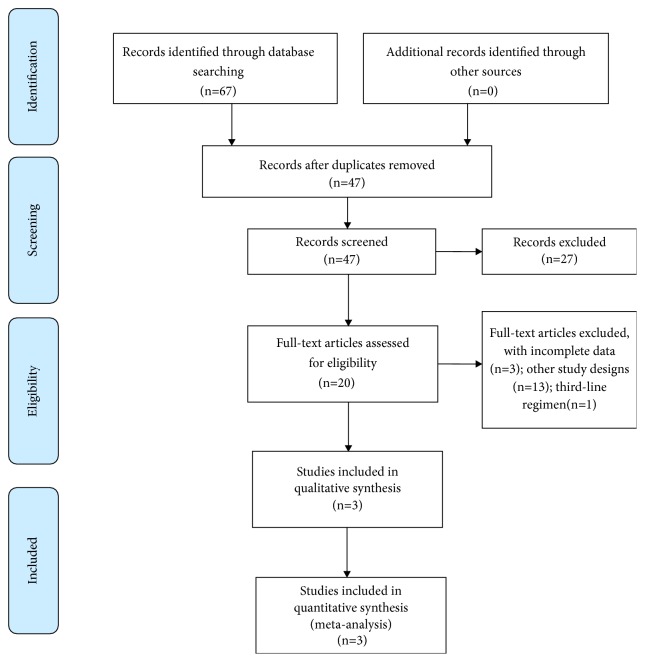
Flow diagram of study identification, screening, inclusion, and exclusion.

The flow diagram of study identification, screening, inclusion, and exclusion is shown in Figure 1. We identified 67 studies in our search of PubMed, Embase, and Cochrane Library databases using the defined terms. Twenty duplicate studies were removed using Endnote software Version X8 and manual screening. Another 27 irrelevant studies were discarded through examining the title and abstract of the articles. After examination of the full text of the remaining 20 articles, we finally selected three with sufficient data for inclusion in this meta-analysis (Table 1). These studies were published between 2016 and 2018, and their enrollment periods ranged from 2012 to 2016. Because vonoprazan was only approved in Japan, all three studies were conducted in Japan. Four hundred and fifty-six patients who received vonoprazan-based triple therapy and 441 who received PPI-based triple therapy were included in this meta-analysis. In all three studies, vonoprazan-based triple therapy consisted of 20 mg vonoprazan, 750 mg amoxicillin, and 200 or 400 mg clarithromycin, twice daily for 7 days. In PPI-based triple therapy, a standard dose of PPI was used instead of vonoprazan. In all three studies, eradication success was confirmed using the UBT at least 4 weeks after completing treatment.

### 3.2. Risk of Bias

**Figure 2:**
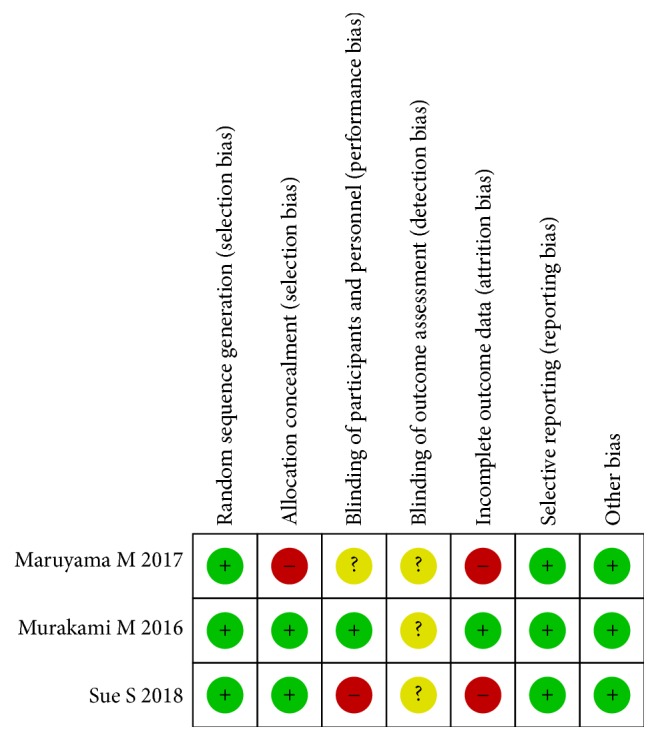
Assessment of bias risk.

Two RCTs showed low risk of bias, but one showed high risk of bias according to the Cochrane Risk of Bias tool (Figure 2).

### 3.3. Efficacy of Vonoprazan-Based versus PPI-Based Triple Therapy

**Figure 3:**
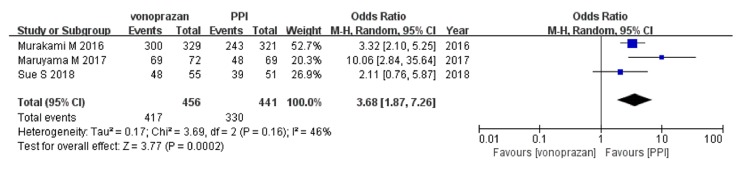
Forest plot of vonoprazan versus PPI-based triple therapy for H. pylori eradication in intention-to-treat analysis. CI, confidence interval; PPI, proton pump inhibitor.

**Figure 4:**
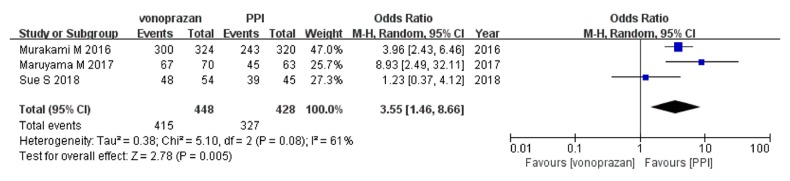
Forest plot of vonoprazan versus PPI-based triple therapy for* H. pylori* eradication in per-protocol analysis. CI, confidence interval; PPI, proton pump inhibitor.

In the ITT analysis (Figure 3),* H. pylori* eradication rate of vonoprazan-based triple therapy was higher than that of PPI-based triple therapy (pooled eradication rates, 91.4% vs 74.8%; OR, 3.68; 95%CI: [1.87–7.26];* P*<0.05). A similar tendency was found in the PP analysis (pooled eradication rates, 92.6% vs 76.4%; OR, 3.55; 95%CI: [1.46–8.66];* P*<0.05) (Figure 4). No significant heterogeneity was identified in the ITT analysis (Cochrane's Q test, df=2,* P*>0.1, I^2^=46%), but significant heterogeneity was identified in the PP analysis (Cochrane's Q test, df=2,* P*<0.1, I^2^=61%).

### 3.4. Safety of Vonoprazan-Based versus PPI-Based Triple Therapy

**Figure 5:**
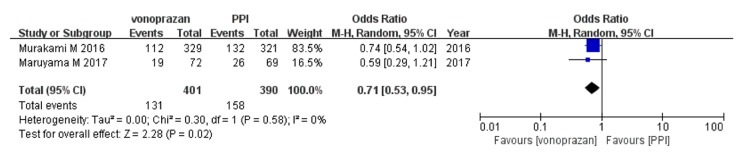
Forest plot of adverse events between vonoprazan versus PPI-based triple therapy. CI, confidence interval; PPI, proton pump inhibitor.

Two studies [22, 23] provided an overall incidence of adverse events and all three studies provided detailed incidence of common adverse events. The overall incidence of adverse events in vonoprazan-based triple therapy was significantly lower than that in PPI-based triple therapy (pooled incidences, 32.7% vs 40.5%; OR, 0.71; 95%CI: [0.53–0.95];* P*<0.05; Cochrane's Q test, df=1,* P*>0.1, I^2^=0%) (Figure 5). To analyze further the safety of the two regimens, we examined the incidence of two common adverse events, namely, diarrhea and dysgeusia. There was no difference in the two regimens (diarrhea: 11.6% vs 18.4%; dysgeusia: 5.7% vs 4.8%;* P*>0.05) (Table 2).

## 4. Discussion

Our meta-analysis demonstrated that vonoprazan-based triple therapy had a higher eradication rate than PPI-based triple therapy as first-line regimen (91.4% vs 74.8%, 95%CI: [1.87–7.26] in ITT analysis; 92.6% vs 76.4%, 95%CI: [1.46–8.66] in PP analysis). These results were consistent with another study that reported eradication rates of >90% for vonoprazan-based triple therapy and <80% for PPI-based triple therapy [25]. According to a report card introduced by Graham to grade* H. pylori* therapy [26], the 91.4% eradication rate in vonoprazan-based triple therapy is good (Grade B), while the 76.4% eradication rate in PPI-based triple therapy is unacceptable (Grade F). Such superiority of vonoprazan-containing triple therapy is because of its faster, stronger, and more stable acid-inhibitory effect [14, 15]. A previous meta-analysis demonstrated that high-dose PPIs seem more effective than standard dose for eradicating* H. pylori* infection in 7-day triple therapy (82% vs 74%, 95% CI:[1.01–1.17]) [27]. Increased gastric pH may drive* H. pylori* to reenter the replicative state and thus become susceptible to antibiotics [28, 29].

Another interesting finding was that vonoprazan-based triple therapy was safer than PPI-based triple therapy, so vonoprazan-based triple therapy would be safe and well-tolerated. If vonoprazan is available and can be afforded by the patients, vonoprazan-based triple therapy should be preferentially recommended, on account of its high efficacy and safety.

Although vonoprazan-based triple therapy was beneficial, significant heterogeneity was still a concern. The heterogeneity may have resulted from the different participants in the included studies. Clarithromycin-susceptible and clarithromycin-resistant subjects participated in the RCTs of Murakami and Maruyama, but only clarithromycin-susceptible patients participated in the RCT of Sue. Clarithromycin resistance is an important factor affecting the efficacy of triple eradication therapy. Many guidelines emphasize that PPI-clarithromycin-containing triple therapy should be rejected if clarithromycin resistance is >15% [3, 4]. In many countries including China and Japan, clarithromycin resistance is >15%. Nevertheless, PPI-clarithromycin-containing triple therapy is commonly used without clarithromycin susceptibility testing because testing is more time-consuming and costlier than empirical treatment. In the presence of clarithromycin resistance, vonoprazan-clarithromycin-containing triple therapy had significantly higher eradication rates as compared to PPI-clarithromycin-containing triple therapy (82.0% vs 40.0%, 95% CI:[3.63–12.86]), and the eradication rate was >80% and an acceptable grade [19, 26]. Vonoprazan-clarithromycin-containing triple therapy may therefore be recommended as empirical treatment when there is no clarithromycin susceptibility test.

Our meta-analysis had several limitations. First, the number of RCTs included was small, and more RCTs are needed to confirm our results. Second, because vonoprazan was only approved in Japan, all studies included in the analysis were performed in Japan, which may have increased selection bias. Our findings may not be generalized to other populations. Third, treatment duration in all RCTs was 7 days; therefore, we cannot assess if vonoprazan-based triple therapy was superior to PPI-based triple therapy other than for 7-days duration. Seven-day triple therapy is not recommended in most guidelines [3, 4]; thus, 14-day triple therapy should be implemented to compare vonoprazan and PPIs. Fourth, all studies enrolled only adult patients, so our results may not be generalized to children. Fifth, all RCTs used triple therapy; thus other eradication regimens, such as bismuth-containing quadruple therapy, concomitant therapy, sequential therapy, and hybrid therapy, should be performed to evaluate if vonoprazan is still superior to PPIs.

## 5. Conclusions

Given the results of our meta-analysis, for the Japanese population, vonoprazan-based triple therapy efficacy is superior to that of PPI-based triple therapy when used as a first-line regimen for* H. pylori *eradication. Additionally, vonoprazan-based triple therapy is better tolerated than PPI-based triple therapy. However, owing to the small number and significant heterogeneity of the included studies and absence of clinical results for other populations, the above conclusions need to be considered with caution.

## Figures and Tables

**Table 1:** Characteristics of studies included in the meta-analysis.

First author	Year	Country	Dosage of vonoprazan/PPIs	Dosage of antibiotics	Duration of treatment	Confirmatory test for eradication	Eradication rates (vonoprazan/PPIs)	Overall incidence rate of adverse events (vonoprazan/PPIs)	Incidence rate of common adverse events (vonoprazan/PPIs)
Murakami K[22]	2016	Japan	vonoprazan 20mg bid; lansoprazole 30 mg bid	amoxicillin 750 mg bid; clarithromycin 200 or 400 mg bid	7 days	^13^C-urea breath test(UBT)	91.2%/75.7% (ITT analysis); 92.6%/75.9% (PP analysis)	34.0%/41.1%	diarrhea:12.5%/15.3% dysgeusia: 4.0%/3.1%
Maruyama M[23]	2017	Japan	vonoprazan 20mg bid; lansoprazole 30 mg bid, rabeprazole 20 mg	amoxicillin 750 mg bid; clarithromycin 200 or 400 mg bid	7 days	^13^C-urea breath test(UBT)	95.8%/69.6% (ITT analysis); 95.7%/71.4% (PP analysis)	26.4%/37.7%	diarrhea:8.3%/14.5% dysgeusia:4.2%/8.7%
Sue S[24]	2018	Japan	vonoprazan 20mg bid; lansoprazole 30 mg, rabeprazole 10 mg, or esomeprazole 20 mg bid;	amoxicillin 750 mg bid; clarithromycin 200 or 400 mg bid	7 days	^13^C-urea breath test(UBT)	87.3%/76.5% (ITT analysis); 88.9%/86.7% (PP analysis)	NA	diarrhea:10.9%/43.1% dysgeusia:18.2%/9.8%

NA: not available; ITT, intention-to-treat; PP, per protocol; PPI, proton pump inhibitor; UBT, urea breath test.

**Table 2:** Occurrence rate of common adverse events between vonoprazan versus proton pump inhibitor-based triple therapy.

adverse events	vonoprazan	proton pump inhibitors	*P* value	heterogeneity test

diarrhea	11.6%	18.4%	0.09	*P*=0.02, I^2^=75%
dysgeusia	5.7%	4.8%	0.65	*P*=0.27, I^2^=23%

## Data Availability

All data was provided in the article.

## Supplementary Materials

Supplementary Materials Appendix S1: detailed search strategy.
