# Supplementary material for: Efficacy and Safety of Vonoprazan-Based versus Proton Pump Inhibitor-Based Triple Therapy for Helicobacter pylori Eradication: A Meta-Analysis of Randomized Clinical Trials
Source: Biomed Res Int. 2019 May 9;2019:9781212. doi: 10.1155/2019/9781212 (PMC6532346; doi:10.1155/2019/9781212)
Supplement: Supplementary Materials — Appendix S1: detailed search strategy. [file 9781212.f1.docx]

**Appendix S1. Detailed search strategy**

**Pubmed**

#1: ((((Vonoprazan[Title/Abstract] OR TAK-438[Title/Abstract] OR TAK438[Title/Abstract] OR Takecab[Title/Abstract])) OR VPZ[Title/Abstract]) OR P-CAB[Title/Abstract]) OR potassium-competitive acid[Title/Abstract]

#2: "1-(5-(2-fluorophenyl)-1-(pyridin-3-ylsulfonyl)-1H-pyrrol-3-yl)-N-methylmethanamine" [Supplementary Concept]

#3: #1 OR #2

#4: (((((((((((("Dexlansoprazole"[Mesh]) OR ((((dexlansoprazole[Title/Abstract] OR Kapidex[Title/Abstract] OR Dexilant[Title/Abstract]))))))) OR Dexrabeprazole[Title/Abstract]) OR ((((("Lansoprazole"[Mesh]) OR ((Lansoprazole[Title/Abstract] OR Lansoprazol[Title/Abstract] OR Lanzor[Title/Abstract] OR lanzo[Title/Abstract] OR lansox[Title/Abstract] OR Prevacid[Title/Abstract] OR Pro Ulco[Title/Abstract] OR Takepron[Title/Abstract] OR Zoton[Title/Abstract] OR "AG 1749"[Title/Abstract] OR "AG-1749"[Title/Abstract] OR AG1749[Title/Abstract] OR Agopton[Title/Abstract]))))))) OR ((((((ilaprazole[Title/Abstract] OR "IY 81149"[Title/Abstract] OR IY81149[Title/Abstract] OR "IY-81149"[Title/Abstract]))) OR "ilaprazole"[Supplementary Concept])))) OR ((((((((tenatoprazole[Title/Abstract] OR benatoprazole[Title/Abstract] OR "TU 199"[Title/Abstract] OR "TU-199"[Title/Abstract] OR Protop[Title/Abstract]))) OR "Tenatoprazole"[Supplementary Concept])))))) OR (((("Rabeprazole"[Mesh]) OR ((rabeprazole[Title/Abstract] OR "E 3810"[Title/Abstract] OR E3810[Title/Abstract] OR Pariet[Title/Abstract] OR Aciphex[Title/Abstract] OR LY307640[Title/Abstract])))))) OR ((((((((omeprazole[Title/Abstract] OR Prilosec[Title/Abstract] OR losec[Title/Abstract] OR rapinex[Title/Abstract] OR zegerid[Title/Abstract] OR omeprazon[Title/Abstract]))) OR "Omeprazole"[Mesh])))))) OR (((("pantoprazole"[Supplementary Concept]) OR ((pantoprazole[Title/Abstract] OR "by 1023"[Title/Abstract] OR "by-1023"[Title/Abstract] OR controloc[Title/Abstract] OR pantoloc[Title/Abstract] OR pantozol[Title/Abstract] OR protium[Title/Abstract] OR protonix[Title/Abstract])))))) OR (leminoprazole[Title/Abstract] OR "NC-1300-O-3"[Title/Abstract])) OR ((((Esomeprazole[Title/Abstract]) OR Nexium[Title/Abstract])) OR "Esomeprazole"[Mesh])

#5:"Proton Pump Inhibitors"[Mesh]

#6:#4 OR #5

#7: (((helicobacter[Title/Abstract] OR pylori[Title/Abstract] OR pyloridis[Title/Abstract] OR "HP"[Title/Abstract] OR Campylobacter[Title/Abstract]))) OR (("Helicobacter"[Mesh]) OR "Helicobacter Infections"[Mesh])

#8: (((randomly[tiab]) OR ((((((trial[ti]) OR clinical trials as topic[mesh:noexp]) OR placebo[tiab]) OR randomized[tiab]) OR controlled clinical trial[pt]) OR randomized controlled trial[pt]))) NOT ((animals[mh] NOT humans[mh]))

#9: #3 AND #6 AND #7 AND #8

**Embase**

#1: 'vonoprazan'/exp

#2: 'vonoprazan':ti,ab OR 'tak-438':ti,ab OR 'tak438':ti,ab OR 'takecab':ti,ab

#3: #1 OR #2

#4: 'omeprazole'/exp OR 'lansoprazole'/exp OR 'pantoprazole'/exp OR 'rabeprazole'/exp OR 'ilaprazole'/exp OR 'dexlansoprazole'/exp OR 'dexrabeprazole'/exp OR 'tenatoprazole'/exp OR 'esomeprazole'/exp

#5: (dexlansoprazole:ti,ab OR kapidex:ti,ab OR dexilant:ti,ab OR dexrabeprazole:ti,ab OR lansoprazole:ti,ab OR lansoprazol:ti,ab OR lanzor:ti,ab OR lanzo:ti,ab OR lansox:ti,ab OR prevacid:ti,ab OR pro) AND ulco:ti,ab OR takepron:ti,ab OR zoton:ti,ab OR 'ag 1749':ti,ab OR 'ag-1749':ti,ab OR ag1749:ti,ab OR agopton:ti,ab OR ilaprazole:ti,ab OR 'iy 81149':ti,ab OR iy81149:ti,ab OR 'iy-81149':ti,ab OR tenatoprazole:ti,ab OR benatoprazole:ti,ab OR 'tu 199':ti,ab OR 'tu-199':ti,ab OR protop:ti,ab OR rabeprazole:ti,ab OR 'e 3810':ti,ab OR e3810:ti,ab OR pariet:ti,ab OR aciphex:ti,ab OR ly307640:ti,ab OR omeprazole:ti,ab OR prilosec:ti,ab OR losec:ti,ab OR rapinex:ti,ab OR zegerid:ti,ab OR omeprazon:ti,ab OR pantoprazole:ti,ab OR 'by 1023':ti,ab OR 'by-1023':ti,ab OR controloc:ti,ab OR pantoloc:ti,ab OR pantozol:ti,ab OR protium:ti,ab OR protonix:ti,ab OR leminoprazole:ti,ab OR 'nc-1300-o-3':ti,ab OR esomeprazole:ti,ab OR nexium:ti,ab

#6: 'Proton Pump Inhibitors'/exp

#7: #4 OR #5OR #6

#8: 'Campylobacter pylori':ti,ab OR 'Helicobacter pylor':ti,ab

#9: 'helicobacter pylori'/exp

#10: #7 OR #8

#11: 'randomized controlled trial'/exp

#12: random* OR blind* OR placebo

#13: #10 OR #11

#14: #3 AND #7 AND #10 AND #13

**Cochrane library**

#1: Vonoprazan:ti,ab,kw or TAK-438:ti,ab,kw or TAK438:ti,ab,kw or Takecab:ti,ab,kw or P-CAB

#2: potassium-competitive acid:ti,ab,kw or VPZ:ti,ab,kw

#3: #1 or #2

#4: MeSH descriptor: [Helicobacter Infections] explode all trees

#5: MeSH descriptor: [Helicobacterpylori] explode all trees

#6: "Helicobacter":ti,ab,kw or "pylori":ti,ab,kw or pyloridis:ti,ab,kw or HP:ti,ab,kw or "campylobacter":ti,ab,kw

#7: #4 or #5 or #6

#8: MeSH descriptor: [Proton Pump Inhibitors] explode all trees

#9: MeSH descriptor: [Esomeprazole] explode all trees

#10: MeSH descriptor: [Rabeprazole] explode all trees

#11: MeSH descriptor: [Omeprazole] explode all trees

#12: MeSH descriptor: [Lansoprazole] explode all trees

#13: MeSH descriptor: [Dexlansoprazole] explode all trees

#14: dexlansoprazole:ti,ab,kw or Kapidex:ti,ab,kw or Dexilant:ti,ab,kw

#15: Dexrabeprazole:ti,ab,kw

#16: Lansoprazole:ti,ab,kw or Lansoprazol:ti,ab,kw or Lanzor:ti,ab,kw or lanzo:ti,ab,kw or lansox:ti,ab,kw or Prevacid:ti,ab,kw or Pro Ulco:ti,ab,kw or Takepron:ti,ab,kw or Zoton:ti,ab,kw or "AG 1749":ti,ab,kw or "AG-1749":ti,ab,kw or AG1749:ti,ab,kw or Agopton:ti,ab,kw

#17: ilaprazole:ti,ab,kw or "IY 81149":ti,ab,kw or IY81149:ti,ab,kw or "IY-81149":ti,ab,kw

#18: tenatoprazole:ti,ab,kw or benatoprazole:ti,ab,kw or "TU 199":ti,ab,kw or "TU-199":ti,ab,kw or Protop:ti,ab,kw

#19: rabeprazole:ti,ab,kw or "E 3810":ti,ab,kw or E3810:ti,ab,kw or Pariet:ti,ab,kw or Aciphex:ti,ab,kw or LY307640:ti,ab,kw

#20: omeprazole:ti,ab,kw or Prilosec:ti,ab,kw or losec:ti,ab,kw or rapinex:ti,ab,kw or zegerid:ti,ab,kw or omeprazon:ti,ab,kw

#21: pantoprazole:ti,ab,kw or "by 1023":ti,ab,kw or "by-1023":ti,ab,kw or controloc:ti,ab,kw or pantoloc:ti,ab,kw or pantozol:ti,ab,kw or protium:ti,ab,kw or protonix:ti,ab,kw

#22: leminoprazole:ti,ab,kw or "NC-1300-O-3":ti,ab,kw

#23: Esomeprazole:ti,ab,kw or Nexium:ti,ab,kw

#24: #8 or #9 or #10 or #11 or #12 or #13 or #14 or #15 or #16 or #17 or #18 or #19 or #20 or #21 or #22 or #23

#25: #3 and #7 and #24
